# Meeting report: transposable elements at the crossroads of evolution, health and disease 2023

**DOI:** 10.1186/s13100-023-00307-4

**Published:** 2023-11-27

**Authors:** Irina R. Arkhipova, Kathleen H. Burns, Katherine B. Chiappinelli, Edward B. Chuong, Clement Goubert, Alba Guarné, Amanda M. Larracuente, E. Alice Lee, Henry L. Levin

**Affiliations:** 1 Josephine Bay Paul Center for Comparative Molecular Biology and Evolution, Marine Biological Laboratory, Woods Hole, MA 02543 USA; 2 Department of Pathology, Dana-Farber Cancer Institute and Harvard Medical School, Boston, MA 02215 USA; 3 Department of Microbiology, Immunology and Tropical Medicine, The George Washington University School of Medicine and Health Sciences, Washington, DC 20052 USA; 4 BioFrontiers Institute and Department of Molecular, Cellular & Developmental Biology, University of Colorado Boulder, Boulder, CO 80309 USA; 5 McGill Genome Centre, Department of Human Genomics, Canadian Centre for Computational Genomics, McGill University, Montréal, Canada; 6 Department of Pharmacy Practice & Science, University of Arizona, Tucson, AZ 85721 USA; 7 Department of Biochemistry and Centre de Recherche en Biologie Structurale, McGill University, Montreal, QC, H3G 0B1 Canada; 8 Department of Biology, University of Rochester, Rochester, NY 14627 USA; 9 Boston Children’s Hospital and Harvard Medical School, Boston, MA 02115 USA; 10 Division of Molecular and Cellular Biology, Eunice Kennedy Shriver National Institute of Child Health and Human Development, National Institutes of Health, Bethesda, MD 20892 USA

**Keywords:** Mobile genetic elements, Mobile DNA, Transposition, Transposons, Retrotransposons

## Abstract

The conference “Transposable Elements at the Crossroads of Evolution, Health and Disease” was hosted by Keystone Symposia in Whistler, British Columbia, Canada, on September 3–6, 2023, and was organized by Kathleen Burns, Harmit Malik and Irina Arkhipova. The central theme of the meeting was the incredible diversity of ways in which transposable elements (TEs) interact with the host, from disrupting the existing genes and pathways to creating novel gene products and expression patterns, enhancing the repertoire of host functions, and ultimately driving host evolution. The meeting was organized into six plenary sessions and two afternoon workshops with a total of 50 invited and contributed talks, two poster sessions, and a career roundtable. The topics ranged from TE roles in normal and pathological processes to restricting and harnessing TE activity based on mechanistic insights gained from genetic, structural, and biochemical studies.

## Introduction

Transposable elements (TEs) are increasingly perceived not only as inevitable constituents of most genomes which persist due to their ability to replicate and spread, but also as active participants in shaping and re-shaping the structural and functional diversity of molecular, cellular, and developmental processes in the host species under normal and pathological conditions and in different environments. To further accelerate progress in this highly dynamic field, Keystone Symposia hosted a three-day meeting “Transposable Elements at the Crossroads of Evolution, Health and Disease” in Whistler, British Columbia, Canada, on September 3–6, 2023. The diversity of host species discussed at the meeting ranged from bacteria to fungi, plants, invertebrates, fish, and mammals. An increasing interest in harnessing the natural ability of transposons to insert into various genomic locations was also evident from presentations given by participants from the biotech industry. The dual nature of TEs as disease-causing mutagenic agents and one of the major driving forces in genome evolution was underscored by the keynote speaker, who made seminal contributions to understanding the roles played by TEs in regulatory evolution across multiple hosts.

## Keynote address

Keynote Speaker Cedric Feschotte, Barbara McClintock Professor of Molecular Biology and Genetics (Cornell University, USA) energized the audience with an inspirational talk using a colorful metaphor of transposon addiction. In contrast to domestication, co-operation, and co-option, which imply that certain parts of TEs are recruited for host functions after losing connection to their original mobilizable units, the concept of directly engaging the actively transposing units into the cellular and developmental processes is relatively new. The Feschotte lab has been investigating the zebrafish retrovirus-like non-autonomous transposon which can still jump in the genome. Knockdown experiments demonstrate that, despite ongoing mobility, its expression is essential for early embryonic development, as manifested by failure to develop proper segmentation in the mesoderm following injection of antisense oligonucleotides. Investigation of the underlying molecular mechanisms of such phenomena observed in zebrafish and other experimental systems is ushering in a whole new direction of research, whereby TEs operating as mobile units become closely intertwined with organismal function to the extent that they can no longer be eliminated without damaging the host.

## In sickness and in health: transposons in disease, immunology, and therapeutics (E. Alice Lee)

Session chair Kathleen Burns opened the first session with the talk given by Vera Gorbunova (University of Rochester, USA) who described her work on transposable elements in aging. First, her lab generated mice with knockdown of the youngest LINE-1 (Long Interspersed Nuclear Element-1, or L1) families, and she discussed the impact of each knockdown on mouse health and lifespan. She then discussed strategies for preventing or reversing age-related LINE-1 activation using *SIRT6* overexpression and pharmacological activators of *SIRT6*, including one derived from brown seaweed.

E. Alice Lee (Boston Children’s Hospital & Harvard Medical School, USA) outlined challenges in identifying somatic retrotransposition events in human tissues, especially in non-dividing cells, such as neurons and cardiomyocytes. Her lab established PTA-HATseq, PCR-based L1-targeted sequencing combined with PTA (primary template-directed amplification), the latest whole genome amplification method, which allows genome-wide somatic L1 insertion profiling at single-nucleotide resolution in single cells. They demonstrated superior performance of PTA-HATseq, detecting ~ 2 times more non-reference insertions in single neurons than existing methods. Their data show that neurons do not show increased somatic retrotransposition with age, whereas epithelial tissues—colon, liver, skin—even postmitotic human cardiomyocytes show age-associated increases, suggesting distinct age-association across different cell/tissue types. They also applied PTA-HATseq to postmortem amyotrophic lateral sclerosis (ALS) and frontotemporal dementia (FTD) patient brain samples. Individual neuronal nuclei were sorted by the status of cytoplasmic TDP-43 protein aggregates, hallmark pathology associated with neurodegeneration in ALS/FTD. ALS/FTD neurons showed more somatic L1 insertions than control neurons, but unexpectedly, more insertions were detected in neurons without TDP-43 pathology, requiring more work to properly interpret them.

Nancy Craig (SalioGen Therapeutics, USA) discussed the pros and cons of different genetic engineering strategies and showed the use and advantages of engineered mammalian DNA transposons derived from the *Myotis lucifugus* transposon (MLT) for targeted gene therapy. These transposons are not expected to cause rearrangements and genomic instability because they do not create double-strand DNA breaks upon payload delivery. MLT has greater capacity for large cargos than other approaches, such as AAV vectors. MLT was specifically engineered to reduce insertional bias, and the whole genome integration profiles showed fewer integration “hotspot” sites and a predilection for insertion into intergenic and intronic regions compared to its un-engineered counterparts. By modifying programmable DNA binding domains, they could boost the accuracy of targeted integrations. Dr. Craig showed multiple use cases of MLT for genetic medicine. The company is working on targeted *CFTR* super-exon insertions to correct recessive mutations in cystic fibrosis, as well as the production of cancer-targeting CAR-T cells. Toward in vivo therapeutics, she presented data on cell-type specific delivery of lipid nanoparticles encapsulating transposase RNA and donor DNA encompassing the *ABCA4* gene, with a length well beyond AAV capacity, into the mouse retina. In summary, these data suggest that MLT provides effective means for targeted gene delivery to enable genetic medicine.

Joshua Dubnau (Stony Brook University, USA) explored positive feedback between endogenous retroviruses (ERVs) and TDP-43 proteinopathy underlying neurodegeneration in ALS/FTD. His lab previously reported that cytoplasmic aggregation of TDP-43 proteins triggers ERV expression. He presented recent data showing that ERV expression can also trigger TDP-43 proteinopathy [1]. They knocked down Drosophila ERV, mdg4 (gypsy), in glia and found reduced propagation of TDP-43 pathology to adjacent glia and neurons. They also found that human ERV (HERV-K) transfected into neuroblastoma cells triggered pathological TDP-43 protein folding. Furthermore, transfected Drosophila S2 cells showed virus-like intracellular transmission of mdg4, which is required for TDP-43 protein pathology in adjacent cells. Together with their previous findings, these data suggest positive feedback between ERV expression and TDP-43 pathology as well as intracellular transmission of ERV and TDP-43 protein aggregates as the cause of non-cell-autonomous toxicity in ALS/FTD.

## Workshop 1: TEs as friends, foes or both (Henry Levin)

This workshop consisted of short talks that captured the important roles TEs have in shaping genome-wide expression, regulating recombination, and generating Mendelian disorders.

The recent advancements in long read DNA sequencing have greatly improved the assembly of genomes with high repeat content. In species such as wheat where TEs occupy over 80% of the genome, comparisons of strains can reveal extensive rearrangements. Dr. Khalil Kashkush (Ben-Gurion University, Israel) identified massive rearrangements between domesticated bread wheat and the wild emmer strains. Indels as large as 1 million bp suggest that such large-scale rearrangements may play a significant role in wheat speciation. By studying the polymorphic TEs within the six homologous genes of hexaploid wheat, Dr. Kashkush presented evidence that TEs inserted in exons or untranslated regions of genes correlated with altered expression [2].

New assemblies of the human genome published by the telomere to telomere (T2T) consortium provide an opportunity to examine the structure of regions with high repeat content. Panpan Zhang (Feschotte lab, Cornell University, USA) presented identification of satellite sequences composed of tandem TEs (SAT-TEs). In the 3574 SAT-TE loci discovered by Dr. Zhang, LINE-1, Alu, and ERV subfamilies were commonly represented. Interestingly, haplotype variation of SAT-TEs revealed high levels of heterozygosity in some individuals. The longest SAT-TE described was a LTR12B locus expanded to 207 units covering 265 kb on the Y chromosome. ENCODE data was used to investigate *cis*-regulatory activity of SAT-TE loci, some of which had characteristics of super-enhancers. The high abundance of SAT-TEs in the human genome and their *cis*-regulatory function suggests these dynamic loci may have significant impact on disease and development.

Session chair Henry Levin (National Institutes of Health, USA) studies LTR-retrotransposons in the fission yeast *Schizosaccharomyces pombe*. Examination of de novo integration showed that insertions often function to increase promoter activity. Earlier, the Levin lab found that de novo insertions can adapt cells to environmental stress by increasing expression of specific groups of genes. Interestingly, the common lab strain of *S. pombe* contains just 13 full-length retrotransposons and Dr. Levin indicated that each is adjacent to a stress response gene including global regulators of oxidative stress and the unfolded protein response. In collaboration with Miguel Zaratiegui (Rutgers University) and Elena Hidalgo (Universitat Pompeu Fabra), Dr. Levin reported that replacing all 13 full-length retrotransposons with single LTRs resulted in sensitivity to peroxide and altered expression of many genes including some adjacent to the TEs. These data suggest that LTR-retrotransposons can provide regulatory input important for resistance to environmental stress.

During development TEs make critical contributions to the expression of gene networks. Jean-David Larouche (Perreault lab, Université de Montréal, Canada) studied thymus development using published human scRNA-seq data. Broad profiles of TEs were found to be expressed in plasmacytoid dendritic cells, providing an association with the stimulation of a proinflammatory microenvironment in the thymus that is favorable to self-tolerance and T-cell development [3].

In addition to influencing gene expression, TEs have the potential to impact other aspects of DNA metabolism. Grace Lee (University of California Irvine, USA) reported that TE abundance is associated with reduced meiotic recombination. To study the nature of this relationship, her lab examined polymorphic TEs for epigenetic marks such as H3K9me2/3 that have the potential to inhibit meiotic recombination. By developing a unique method, pooled PacBio sequencing, for mapping crossover events in two Drosophila inbred strains with different TE profiles, they found that TEs with enrichment of repressive marks were associated with reduced rates of crossovers, indicating that TEs can be modifiers of meiotic recombination.

The contributions of TEs to their host go beyond modifying existing sequences. Sonya Widen (Burga lab, Institute of Molecular Biotechnology, Austria) described her discovery that Maverick TEs can function to horizontally transfer certain cargo genes between divergent species [4]. While horizontal gene transfer (HGT) has been observed across all major eukaryotic lineages, the responsible mechanisms are unclear. Mavericks/Polintons are ancient virus-like TEs that encode many proteins including an integrase, a DNA polymerase, and major and minor capsid proteins. Dr. Widen found that in nematodes, Mavericks also code for a putative fusogen, structurally similar to viral fusogens. By studying selfish toxin-antidote elements in *Caenorhabditis briggsae,* she found that two nematode gene families, wosp proteases and krma kinases, were acquired as cargo by Mavericks and transferred between different nematode species on a global scale. This work reveals how HGT can drive genetic incompatibilities in natural populations.

Although it is widely understood that TE insertions in early development or in the germline can result in spontaneous cases of disease such as Factor VIII hemophilia, there has been no systematic measure of TE-generated Mendelian disease. To address this question, Boxun Zhao (Lee and Yu labs, Boston Children’s Hospital & Harvard Medical School, USA) profiled retroelement insertions in whole-genome sequences (WGS) for 237 patients with ataxia telangiectasia. Analysis of the disease-causing gene *ATM* revealed one exonic and 12 intronic loss-of function insertions, accounting for 2.1–5.5% of the cohort. This provides an initial estimate of the TE contribution to Mendelian disease [5]. Importantly, Dr. Zhao also found that some antisense oligonucleotides showed therapeutic potential in correcting mis-splicing events caused by TE insertions.

Another study of a neurological disease was presented by Alexandra Whiteley (University of Colorado, Boulder), who studies *PEG10*, a domesticated TE gene that is necessary for placentation. Elevated expression of *PEG10* has been implicated in a neurodevelopmental disease, Angelman’s syndrome. Earlier, she found that a proteasome shuttle factor, UBQLN2, facilitates degradation of the gag-pol from *PEG10*. Importantly, mutations in *UBQLN2* cause the neurodegenerative disease ALS. Dr. Whiteley now finds that a nucleocapsid-like fragment of PEG10 localizes in the nucleus and alters expression of genes involved in axon remodeling [6]. Additionally, PEG10 levels are elevated in spinal cord tissue from ALS patients relative to healthy controls. Her lab is currently pursuing the hypothesis that PEG10 has activity that contributes to ALS through the regulation of neuronal gene expression.

## Genomic battlegrounds: evolutionary conflicts and arms races in the mobilome (Amanda Larracuente)

Session chair Harmit Malik (Fred Hutchinson Cancer Center, USA) kicked off the session with a discussion of host-virus arms races, which drive the rapid evolution of viral and antiviral proteins. He focused on one rapidly evolving primate protein involved in retroviral restriction: TRIM5α. This work, led by former postdoc Jeannette Tenthorey, used deep mutational scanning to study how mutations in the region of TRIM5α involved in binding retroviral capsids, the ‘v1’ loop, affect its ability to restrict diverse retroviruses [7]. His talk highlighted two distinct and surprising strategies deployed by primate TRIM5 proteins in this antivirus-virus arms race. The first strategy is mutational resilience, where there are many ways to restrict HIV-1 through single amino acid changes in the v1 loop that affect charge without compromising existing functions. Unpublished work highlighted a second strategy in which indel mutations can also be an important source of evolutionary innovation for retroviral protection.

Zhao Zhang (Duke University, USA) presented his lab’s recently published work on how transposable elements hijack processes in the female Drosophila gonad to integrate into oocyte DNA and the role of extrachromosomal circular DNAs in this process [8]. Dr. Zhang discussed how the alt-EJ pathway is important for circularizing LTR retrotransposons to complete second strand synthesis, which is important for integration. The second part of his talk was on their early-stage and unpublished work on immunogenic transposons and potential applications in cancer immunotherapy and anti-aging therapies.

Amanda Larracuente (University of Rochester, USA) discussed centromere evolutionary dynamics among four closely related Drosophila species: *D. melanogaster* and the simulans clade (*D. sechellia*, *D. simulans*, and *D. mauritiana*). Centromeres are essential structures for chromosome segregation during cell division but occur in rapidly evolving genome regions rich in repeats. Combining comparative genomics and cytology, Larracuente’s lab discovered dramatic centromere reorganization involving recurrent shifts between retroelements and satellite DNAs over short evolutionary timescales. They also revealed the recent origin of telocentric chromosomes in *D. sechellia*, where X and 4th chromosome centromeres now sit on telomere-specific retroelements [9]. This rapid centromere turnover is consistent with genetic conflicts in the female germline and has implications for centromeric DNA function and karyotype evolution.

Caroline Langley (Fred Hutchison Cancer Center, USA) gave a short talk on host-virus arms races between rapidly evolving host APOBEC3 proteins involved in restricting retroviruses and viral-encoded infectivity factor (Vif). Langley’s focus was on the so-called arms race interface between human A3G and HIV-1 Vif, originally defined by patterns of molecular evolution and recently confirmed through cryo-EM structure [10]. They show that sequence diversity in Vif at the arms race interface reflects A3G diversity in hominids and old-world monkeys. Langley also discussed unpublished work using deep mutational scanning to explore residues important for Vif function and evidence that the overlap between Vif and the HIV-1 integrase constrains Vif evolution.

Josien van Wolfswinkel (Yale University, USA) gave a short talk on transposable elements and regulation of planarian stem cells (neoblasts). Planarians are well studied models for tissue regeneration and the expression of PIWI proteins in neoblasts is important for regulating transposons and the regeneration process. Van Wolfswinkel discussed how the loss of the nuclear PIWI protein SMEDWI-2 has a tissue-specific effect on transposable element de-repression, leads to changes in chromatin landscapes and ultimately, the failure to properly undergo cell differentiation [11]. Van Wolfswinkel’s further work reveals multiple ways that transposons influence stem cell function and survival.

## Epigenomics and epitranscriptomics of transposition and interactions with the environment (Irina Arkhipova)

The opening talk by Haruhiko Siomi (Keio University School of Medicine, Japan) reinforced the paradigm-shifting concept outlined in the keynote address that TEs do not need to be converted into single-copy domesticated genes to become integral components of the host pathways and developmental processes. Studies of the murine retrovirus MERV-L revealed that it regulates a critical developmental timing window that precedes zygotic genome activation (ZGA). During the first cleavage divisions, MERV-L is transiently upregulated, while its knockdown at this stage induces a prolonged 2-cell-like state that results in developmental defects, embryo degeneration and death. Surprisingly, neither MERV-L* trans-*acting RNA nor MERV-L-encoded proteins are required for promoting the transition from totipotency to pluripotency, shifting the attention on the changes in chromatin state that cause improper expression of two-cell-specific genes [12].

Magnus Nordborg (Gregor Mendel Institute, Austria) presented a global overview of TE dynamics in 500 sequenced natural accessions of *Arabidopsis thaliana* derived from distinct geographical regions. He reported strong geographic variation in TE load between accessions, underscored the role of TEs as major contributors to structural and epigenetic variation, and discussed the principal factors contributing to TE population dynamics, including genetic polymorphisms in different components of the epigenetic silencing machinery, and the influence of environmental factors such as temperature. TE fragments within transcriptional units were shown to induce silencing at the corresponding transcribed loci [13].

Requirements for establishment of piRNA-based epigenetic silencing were uncovered by Alexei Aravin (Caltech, USA), whose lab studied a *Gal4*-driven GFP insertion into the major *Drosophila melanogaster* germline dual-strand piRNA cluster 42AB. Promoter inducibility was used to assess the ratio of canonical transcription to non-canonical transcription, which is dependent on the RDC complex (Rhino-Deadlock-Cutoff). The team found that establishment of piRNA clusters is a much less straightforward process than previously thought, and requires maternal transmission of the cytoplasmic siRNA signal over several generations for robust establishment of non-canonical transcription and chromatin-based piRNA silencing, regardless of TE placement within any pre-existing piRNA clusters [14].

The short talk by Sharon Schlesinger (Hebrew University, Israel) focused on the role of new players Smarcad1 and the histone variant H3.3 in reinforcing the familiar system of heterochromatin-based retroviral silencing in mouse ES cells based on the master regulator Trim28/KAP1, KRAB-Zn-fingers and SETDB1 H3K9 methyltransferase. She introduced a concept of “dynamic retrochromatin”, according to which the deposition of the replication-independent H3.3 helps to maintain prolonged silencing of retroviral sequences and relies on Smarcad1, an ATP-dependent nucleosome exchange factor which controls nucleosome disassembly and reassembly. Depletion of Smarcad1 led to retroviral de-repression and failure to recruit Trim28 and H3.3 to the silenced loci [15].

Jeffrey Hyacinthe (Bourque lab, McGill University, Canada) in his short talk unveiled a global overview of the human epigenome as it relates to TEs, fueled by the wealth of data from eight consortia fed into the International Human Epigenome (super) Consortium (IHEC) (https://ihec-epigenomes.org/). Spanning the best-characterized modifications of the H3 histone in 5472 ChIP-seq samples across 60 cell types, the analyzed dataset includes the marks typically associated with transcriptional activation at promoters or enhancers (H3K4me1, H3K4me3, H3K27ac, H3K36me3) as well as with repression (H3K9me3, H3K27me3). Initial analysis reveals that some TE families and subfamilies are disproportionally enriched in certain cell types, while others can be silenced across multiple cell types.

In contrast to the global analysis of human epigenomes, Cristina Tufarelli (University of Leicester, UK) focused her short talk on a single antisense promoter of a specific human L1 copy, which is linked to silencing of an adjacent metastasis suppressor gene and drives expression of a colon-specific chimeric transcript joining L1 ORF0 to exons 2 and 3 of *GNGT1*, a gene which is normally expressed in the eye. Notably, both chimeric RNA and GNGT1 protein are enriched in colon cancer tumors, including premalignant tumors; chimeric RNA expression levels increase with age and its forced expression transforms non-neoplastic colon epithelial cells. These findings underscore the potential of L1 antisense promoters to drive ectopically expressed isoforms, some of which may participate in carcinogenesis and elevate the risk of malignant transformation.

The concluding talk by session chair Irina Arkhipova (Marine Biological Laboratory, USA) placed the emphasis on the host response to environmental stresses, such as amino acid starvation or the presence of certain antibiotic agents, and on the involvement of domesticated reverse transcriptase-like genes (*rvt*) in helping bacteria, fungi and rotifers to overcome such stresses. The single-copy *rvt* genes represent a distinct type of reverse transcriptases (RTs) which are widespread in free-living, mostly soil-dwelling, organisms, evolve under purifying selection, and are often subject to horizontal transfers. The catalytic activity displayed by rvt proteins involves non-templated polymerization, and intact catalytic residues are required for alleviating the effects of stress.

## Transposon domestication and co-option at the DNA, RNA and protein levels (Edward Chuong)

Alan Lambowitz (University of Texas at Austin, USA) presented a large body of work on the characterization of bacterial reverse transcriptases (RTs), including mobile group II intron RTs. He discussed work showing that mobile group II introns encode active RTs and elucidated group II intron retromobility mechanisms. He also presented work biochemically and structurally characterizing group II intron RTs and their use for biotechnological applications, including targetrons, the first RNA-guided gene targeting vectors, and TGIRT-seq, a high-throughput RNA-sequencing method that utilizes the advantageous biochemical properties of mobile group II intron RTs for comprehensive RNA profiling from small amounts of starting material, including tumors and blood [16]. Functions of domesticated RTs include double-strand break repair by group II intron-like RTs [17] and incorporation of RNA spacers into CRISPR arrays via non-templated tailing by RT-Cas1 proteins [18].

Miguel Branco (Blizard Institute, Queen Mary University of London, UK) presented his work investigating ERV-derived enhancers in the placenta. He showed how ERVs provide functional species-specific enhancers in both mouse and human trophoblast cells, potentially contributing to species-specific biological differences affecting placental development and function [19]. He also reported CRISPR deletion experiments showing that some ERVs regulate genes involved in placental disorders in human. His work implicates ERV regulatory activity as a potentially important factor contributing to pregnancy outcomes.

Session chair Edward Chuong (University of Colorado Boulder, USA) presented his work on TE exonization affecting the evolution of immune gene isoforms. Long-read RNA-seq data enabled the discovery of novel or poorly characterized TE exonization events that show high levels of expression, in contrast to the general assumption that they are poorly expressed [20]. He presented functional characterization of one example affecting the type I interferon receptor alpha/beta 2 (*IFNAR2*), which generated a broadly expressed truncated isoform that acts as a decoy receptor.

Rachel Cosby (MacFarlan Lab, NIH/NICHD, USA) presented her work on THAP7, a putative transcription factor derived from a *P-*element-like transposase fusion that is conserved in all jawed vertebrates. She described CRISPR knockout, RNA-seq, and ChIP-Seq characterization of *THAP7* in human, mouse, and zebrafish cells. In zebrafish, *THAP7* knockout was fatal, while in mouse, *THAP7* knockouts show sex-dependent behavioral defects. Her work highlights a role for THAP7 in vertebrate development and human intellectual disability, and provides an exciting comprehensive study of the origin and function of host-transposase fusion genes.

Azra Lari (Glaunsinger lab, UC Berkeley, USA) presented work exploring the role of B2 SINE retrotransposon activation in modulating mRNA isoform switching during gammaherpesvirus infection. Using long-read RNA-seq, she identified hundreds of host isoform-switching events dependent on virus-activated B2 SINEs and determined that these switching events were enriched for transcripts that encode innate immune factors. This work points to a mechanism that drives host-isoform switching during viral infection through the co-option of B2 SINE elements [21].

## Transposable elements in cancer (Katherine Chiappinelli)

Session chair Dixie Mager (BCCRC, Canada) opened the session with the talk by Kathleen Burns (Dana Farber Cancer Institute & Harvard Medical School, USA). She presented work demonstrating that the LINE-1 open reading frame 1 protein (ORF1p) is overexpressed in cancers and precursor lesions, while it has lower expression in normal tissues. ORF1p is highly expressed in solid tumors including ovarian, colon, uterine, gastro-esophageal, pancreatic, and breast cancers, among other malignancies. Dr. Burns described design and optimization of a single molecule array (Simoa) assay able to detect ORF1p levels in the blood, with significantly higher ORF1p in blood of cancer patients as compared to healthy controls [22]. This biomarker is perhaps especially promising for ovarian cancer, which is often detected at Stage III/IV when the prognosis is dire and for which there is no reliable screening test. Dr. Burns also presented studies showing that LINE-1 retrotransposition in human cells causes DNA damage and slows replication fork progression, building on earlier work [23]. These findings implicate LINE-1 as a both a marker and a genome mutator in human cancers, support the use of LINE-1 ORF1p as a means to detect or monitor disease, and raise the possibility of leveraging ORF2p activities for cancer therapeutics.

John Moran (University of Michigan Medical School, USA) presented studies describing the epigenetic silencing of reporter genes integrated into the genomic DNA of a human embryonic carcinoma (hEC) cell line by human LINE-1 (L1) retrotransposition. LINE-1 reporter gene silencing (also termed L1-REPEL) appears to be specific to retrotransposons that mobilize via TPRT (target-primed reverse transcription), and occurs either during or immediately after L1 integration. The treatment of hECs containing silenced L1-enhanced green fluorescent protein (L1-EGFP) integration events with the histone deacetylase inhibitor trichostatin A (TSA) led to a rapid activation of L1-EGFP reporter gene expression, whereas subsequent TSA removal led to the reestablishment of L1-EGFP reporter gene silencing, suggesting evidence of epigenetic memory. Dr. Moran then described how genome-wide CRISPR/Cas9-based genetic screens led to the identification of *NF2*, a tumor suppressor gene, as a candidate host factor necessary for L1-REPEL. Subsequent experiments revealed that *NF2* appears to be necessary for the initiation, but not maintenance, of L1-REPEL. In sum, these findings suggest that *NF2* plays a critical, albeit perhaps indirect, role in combatting unabated L1 retrotransposition in a hEC cell line that serves as a proxy for early stages of human development.

Ting Wang (Washington University in St. Louis, USA) presented work on how transposable elements shape three-dimensional chromatin interactions in mammalian cells [24]. TEs provide binding sites for proteins that shape chromatin architecture, including CTCF. These protein-binding sites can both shape *cis*-interactions between enhancer and promoter regions to affect transcription and create boundary regions in between to segregate chromatin states. One example is an L1MC1-anchored loop that is human-specific that is lost upon deletion of the TE. These findings shed light on how transposable elements alter species-specific chromatin structure to control gene expression in mammals.

Katherine Chiappinelli (George Washington University, USA) presented work on epigenetic regulation of TEs in cancer genomes. Dr. Chiappinelli and colleagues demonstrated that the tumor suppressor P53 transcriptionally activates TEs, especially LTR and Alu elements, in cancer cell lines. Combining P53 activation with inhibition of DNA methylation increases TE transcriptional activation and hot spot mutant P53 cell lines exhibited differential TE regulation [25]. Beyond transcriptional regulation, the ADAR1 enzyme edits TE RNA to inhibit its binding to the MDA5 cytosolic sensor and induction of a downstream interferon response. When ADAR1 is inhibited alongside inhibition of DNA methylation in ovarian cancer cell lines and murine models, type I interferon is activated, increasing immune signaling to reduce tumor burden and significantly improve survival [26]. These data demonstrate strategies to activate TE transcription to induce an anti-tumor immune response in ovarian cancer.

Martin Taylor (Massachusetts General Hospital & Harvard Medical School, USA) presented the structure of the LINE-1 ORF2 protein in different states. High resolution structures were determined for the ‘core’ ORF2p (encompassing RT and novel ‘tower’ and ‘wrist’ domains, involved in retrotransposition and RNA binding, respectively), by X-ray crystallography and cryo-EM in active conformations, bound to template RNA-only, and in apo form. The apo form is unstable and in a ‘thumbs up’ conformation can bind RNA, which may explain L1 *cis*-preference. Lower resolution structures of the full-length ORF2p show dynamic conformations of the N-terminal endonuclease (EN) and C-terminal domains that appear to allow ‘open’ and ‘closed ring’ states. The ORF2p structure is more similar to bacterial group II intron and other non-LTR RTs than to viral and LTR RTs. Biochemically, ORF2p is highly active and processive as both RT and DNA-directed polymerase but has limited RdRP activity. Unexpectedly, it also is efficiently primed by RNA duplexes and hairpins, including an Alu-derived RNA hairpin. In cells, ORF2p can synthesize inflammatory RNA:DNA hybrids in the cytosol, which are RT- but not EN-dependent, ruling out an origin from ‘nuclear ejection’ of TPRT intermediates. ORF2p structure explains the potencies of existing nucleoside inhibitors and the lack of inhibition by non-nucleoside RT inhibitors (NNRTI) of HIV, and provides a basis for rational design of specific LINE-1 RT inhibitors for novel therapies against cancer and autoimmune diseases.

The short talk by Siyu Sun (Greenbaum lab, Memorial Sloan Kettering Cancer Center, USA) presented a comprehensive study of TEs in 214 total RNA-seq samples with 66 matched WGS data from rapid autopsies of patients with pancreatic ductal adenocarcinoma [27]. In this cohort there were several examples of LINE-1 somatic insertions during tumor evolution. In addition, newly evolved SINE elements, which are more immunostimulatory, exhibited a stronger association with RIG-I like receptor mediated type I interferon signatures, and are strongly repressed in samples with high L1 ORF1p expression and high L1 mobility. The RNA editing enzyme ADAR1, which changes the structure of immunogenic SINEs, was negatively correlated with the type I interferon signature and also LINE-1 insertions. Lastly, patients with mutations in the p53 tumor suppressor exhibited less ADAR1 RNA editing. These data shed light on the co-evolution of tumors and the tumor microenvironment to regulate immunostimulatory TE RNA.

## Workshop 2: Bioinformatic and multi-omic tools (Clément Goubert)

This workshop, featuring a series of short talks, was devoted primarily to tool development in the most challenging areas of TE research, such as structural variation and methylome analysis in populations and single-cell analysis of TE transcripts and their isoforms. It was chaired by Arian Smit (Institute for Systems Biology, USA).

Clément Goubert (Bourque lab, McGill University, Canada, and Wheeler lab, University of Arizona, USA) presented GraffiTE, a workflow dedicated to study TE insertion polymorphisms from a wide range of data (genome assemblies, long and short-read sets, and catalog of structural variants). The method introduces graph genomes to represent TE variation resulting in enhanced estimation of allele frequencies, and is applicable to models with no prior knowledge on TEs [28].

Xiaoyu Zhuo (Wang lab, Washington University at St Louis, USA), harnessing the high-quality data of the human pangenome reference consortium, demonstrated how PacBio CLR (continuous long reads) can be used to infer the methylation state of TEs across populations. Showing high correlation between the methylation status inferred from CLR reads and bisulfite sequencing, Dr. Zhuo showed that (i) most TE insertions are hypermethylated in the human population, (ii) Alu insertions in hypomethylated CpG islands have also low level of methylation, and (iii) there is a 3–4% increase in methylation around L1 and Alu insertions.

Opening a series of talks on single-cell RNA-seq analysis of TEs, Jun Ding (McGill University, Canada) introduced MATES (Multi-mapping Alignment for TE Expression quantification in Single-cell), a deep learning method to estimate a read’s multi-mapping probability based on the genomic context of each TE locus. This approach allows locus-specific quantification, showing improvement of cell clustering based on TE expression, and can be also used with single-cell ATAC-seq experiments.

Tackling the same question with alternative algorithms, Matthew Bendall (Weill Cornell Medicine, USA) presented Stellarscope, a single-cell version of TElescope, which uses Expectation-Maximisation to quantify TE expression. Notably, Dr. Bendall described an original approach using adjacency graphs to remove PCR duplicates, and demonstrated how different pooling modes available in the software help with the assignment of multi-mapping reads.

Beyond their canonical expression, TEs can also be co-opted as alternative gene promoters. To identify and quantify such TE-derived transcripts across different tissues, cell types, or disease states, Bo Zhang (Washington University at St Louis, USA) developed TSRdetector, which operates at single-cell resolution. Dr. Zhang’s application of TSRdetector revealed that ~ 15% of human protein coding genes show isoforms initiated by a TE.

Bimala Acharya (Anderson lab, Iowa State University, USA) unveiled a method to detect genes trans-duplicated via rolling-circle elements of the *Helitron* superfamily. *Helitrons* are particularly challenging to identify, as they do not bear typical TE signatures such as target site duplications or terminal repeats. To address this challenge, the approach searches for clusters of non-syntenic gene fragments, flanked by ATC (5′) or CTRR (3′) motifs, indicative of the *Helitrons*’ termini.

Next, Anubhuti Mathur (Tessera Therapeutics, USA) described the company’s efforts to harness non-LTR retrotransposons for precise insertion of long sequences in the context of therapeutic genome editing. A bioinformatic search across all kingdoms of life yielded thousands of candidate elements, which were screened for activity in human cells. To further maximize efficacy, ancestral sequence reconstruction (ASR) was implemented to generate putative ancestral sequences to enhance the integration of transgene RNA. High-throughput screening of the ASR library highlighted the important role of engineered UTR sequences to increase gene writing efficiency [29].

In a closing talk, Asiya Gusa (Duke University, USA) presented her work on the environmental pathogenic fungi from the genus *Cryptococcus*, which can cause fatal meningitis, particularly in immunosuppressed subjects. Though mainly clonal, *Cryptococcus* display remarkable adaptation to heat stress, which occurs at human body temperature. In experiments at 37 °C, *Cryptococcus* showed increased drug resistance, driven by TEs as the primary source of mutation [30]. In the context of global warming, Dr. Gusa’s lab is now focusing on elucidating the molecular mechanisms associated with this emerging threat.

## Structural and mechanistic underpinnings of transposition across the kingdoms of life (Alba Guarné)

Session chair Orsolya Barabas (University of Geneva, Switzerland) opened the session by discussing conjugative transposons carrying antibiotic resistance genes – a significant health challenge due to their linkage to the emergence of multidrug-resistant pathogens. Her talk built upon comprehensive work from her laboratory using tyrosine recombinases to annotate mobile genetic elements in prokaryotes [31]. She described the biochemical and structural characterization of a tyrosine recombinase from one of these elements, revealing a complex architecture essential for function.

Phoebe Rice (University of Chicago, USA) continued the site-specific recombinase theme by describing structure-function studies of small and large serine recombinases and the different mechanisms by which their reaction directions are controlled. These recombinases are carried by the SCCmec mobile genetic elements conferring methicillin resistance to “MRSA” strains of *Staphylococcus aureus*, which represent a significant public health problem worldwide.

Jeff Miller (UCLA, USA) switched gears to discuss diversity-generating retroelements (DGRs) in bacteria and how they accelerate protein evolution through mutagenic retrohoming [32]. DGR-encoded reverse transcriptases perform adenine-specific mutagenesis to diversify the C-termini of target proteins. Reverse transcription is self-primed by the RNA template, however the basis for misincorporation at adenines is still not known. He presented the work from his lab on how Bacteroides DGRs, enriched in the human gastrointestinal microbiome, diversify target genes in vitro, in gnotobiotic mice, and in humans.

The session ended with two short talks on bacterial transposons from the Tn7 family. Elizabeth Kellogg (Cornell University and St. Jude Children’s Research Hospital, USA) discussed cryo-EM work from her laboratory on one of the programmable CRISPR-Associated Transposons (CASTs) from this family [33]. The CAST type V-K element is of particular interest for genome-editing applications due to the minimal size of its transposon machinery. She discussed how this element assembles a functional transpososome and ensures the spacing between the target and insertion sites.

Alba Guarné (McGill University, Canada) presented the biochemical characterization and cryo-EM structure of the target-site selection complex from the prototypical Tn7 element. Building upon previous work from her laboratory describing the structure of the AAA+ ATPase adaptor protein TnsC [34], they went on to characterize the TnsD-mediated target site selection complex. The work reveals that the assembly of this complex is a slow, stepwise process, in turn informing how the assembly process may also have a role in transposition immunity.

Closing remarks were delivered by Irina Arkhipova, who briefly outlined the history of the Keystone TE meetings dating back to 1991, and encouraged members of the community to attend upcoming in-person and/or hybrid TE meetings. The EMBO Workshop “The mobile genome: genetic and physiological impacts of transposable elements” took place in Heidelberg, Germany, on November 8–11, 2023. The International Congress on Transposable Elements (ICTE) in St. Malo, France will again welcome participants on April 20–23, 2024. The biannual Cold Spring Harbor meeting on Transposable Elements in Cold Spring Harbor, NY will follow on October 15-19, 2024, and the next FASEB Science Research Conference on mobile DNA is planned for the summer of 2025.

## Outcomes and future directions

The main outcome of the meeting was the establishment and maintenance of scientific interactions between researchers who would not have the time and opportunity to do so in their routine schedules. Such encounters have the potential to catalyze generation of non-trivial ideas and cross-fertilization of streamlined approaches established within narrowly specialized fields. The traditional free exchange of unpublished data is now greatly facilitated by the availability of preprints, which we opted to cite in this report for easier reference. The highly condensed three-day format minimized the available free time, but maximized the opportunities for younger researchers to have their science heard.

In the future, we are expecting major advancements in the TE field to be driven by cutting-edge technologies such as determination of complete linear and spatial structures of eukaryotic chromosomes unveiling their true repetitive content, genetic manipulation of multicopy TEs validating their roles in the host biology, single-cell analysis techniques, advanced imaging and structural analysis of molecules and macromolecular complexes, and large-scale data processing with machine learning algorithms. Practical applications are expected to capitalize on TE potential as disease markers and causative agents, as well as vehicles for targeted insertion of large payloads.

The post-pandemic reinvigoration of the mobile DNA field is manifested in a series of upcoming high-profile in-person and/or hybrid international meetings scheduled to occur in the coming two years on both sides of the Atlantic, relieving the travel burden to some extent. It is rewarding to see that the field is constantly attracting new organizers and new participants, helping to jump-start new collaborations and exchange ideas that will yield breakthrough discoveries in the years to come.

## Data Availability

Data sharing not applicable to this article as no datasets were generated or analysed during the current study. Any unpublished data mentioned herein cannot be cited without presenters’ permission.

## Abbreviations

AAV: Adeno-associated virus
ADAR: Adenosine deaminase acting on RNA
ALS: Amyotrophic lateral sclerosis
ChIP: Chromatin immuno-precipitation
CLR: Continuous long reads
CRISPR: Clustered regularly interspaced short palindromic repeats
cryo-EM: Cryogenic electron microscopy
DGR: Diversity-generating retroelements
ENCODE: Encyclopedia of DNA elements
ERV: Endogenous retrovirus
ES cells: Embryonic stem cells
FTD: Frontotemporal dementia
GFP: Green fluorescent protein
HDAC: Histone deacetylase
HGT: Horizontal gene transfer
LINE-1, L1: Long interspersed nuclear element 1
LTR: Long terminal repeat
piRNA: PIWI-interacting RNA
PIWI: P-element-induced wimpy testes
RdRP: RNA-dependent RNA polymerase
RT: Reverse transcriptase
scRNA-seq: Single-cell RNA sequencing
T2T: Telomere to telomere
TE: Transposable element
TPRT: Target-primed reverse transcription
UTR: Untranslated region
WGS: Whole-genome sequencing
ZGA: Zygotic genome activation
